# Evidence of bovine immunodeficiency virus: A molecular survey in water buffalo populations of Iran

**DOI:** 10.1002/vms3.872

**Published:** 2022-07-04

**Authors:** Haniyeh Keshavarz, Ali Mohammadi, Solmaz Morovati

**Affiliations:** ^1^ Department of Pathobiology School of Veterinary Medicine Shiraz University Shiraz Iran

**Keywords:** BIV, buffalo, PCR, *pol* gene, prevalence

## Abstract

**Background:**

Bovine immunodeficiency virus (BIV) is a member of the Retroviridae family causing a progressive lifelong infection in cattle and buffaloes.

**Objective:**

Despite the worldwide distribution of the virus, the studies concerning the prevalence of BIV in buffalo populations have not been conducted in Iran as yet.

**Methods:**

The BIV proviral DNA was surveyed in 120 whole blood samples of water buffaloes in southwestern Iran. Nested PCR was employed to amplify a 298‐bp fragment of the *pol* gene. The BIV *Pol* sequence was detected in 9.1% of the samples. Among PCR‐positive samples, two amplified fragments were confirmed by nucleotide sequencing.

**Results and conclusions:**

The studied sequences were completely identical to each other and had more than 98%–99% nucleotide homology to R‐29 and HXB3 sequences previously deposited in GenBank. Some point mutations that caused coding substitutions were observed in the studied isolates, compared to other strains. A phylogenetic tree was generated based on the BIV *Pol* nucleotide sequences reported from other countries. All the BIV strains originated from a unique main cluster and then separated from each other over time. This is the first report on the molecular detection of BIV infections in water buffalo populations in Iran. The wide distribution of BIV in different countries including Iran indicates the importance of the infection as it relates to animal health. Although buffaloes show greater resistance to diseases, they should be considered a health risk to cattle. Furthermore, BIV has negative effects on buffalo milk production and can predispose them to secondary infections. Hence, the findings of this study can advance our understanding of the occurrence of BIV infection in Iran, which can play an important role in the distribution of the disease worldwide.

## Graphical abstract

In the present study, the BIV proviral DNA was detected in whole blood samples of water buffaloes in southwestern Iran for the first time using nested‐PCR. Some positive samples were then confirmed by nucleotide sequencing and subjected to subsequent phylogenetic analyses. The studied sequences were completely identical to each other and had more than 98–99% nucleotide homology to R‐29 and HXB3 sequences previously deposited in GenBank.

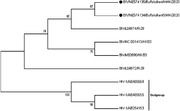

## 1 INTRODUCTION

The bovine immunodeficiency virus (BIV), a lentivirus belonging to the Retroviridae family, causes a progressive and persistent infection in cattle (Gonda et al., 1987). The genome of the virus consists of two copies of a single‐stranded, positive‐sense RNA that can integrate into the cellular DNA. The first isolate of BIV was identified in 1969 from an adult dairy cow with lymphocytosis, lymphadenopathy, weakness, emaciation and central nervous system lesions. BIV is genetically and antigenically related to human immunodeficiency virus type I (HIV‐I) and simian immunodeficiency virus, so it is used as an animal model for the study of other retroviruses (Garvey et al., 1990; Gonda et al., 1987).

The infected animals did not show signs of severe illness. However, the haematological profile of the BIV‐infected cattle indicates lymphocytosis, lymphadenopathy and monocyte and neutrophil dysfunctions. Skin lesions unresponsive to therapy and meningoencephalitis are also reported in infected cattle. It is supposed that BIV infection may be related to a decrease in milk yield and weight loss in infected animals and promote secondary infections such as BLV (Meas, Ohashi, et al., 2000; Meas, Seto, et al., 2000; Nikbakht Brujeni et al., 2010).

BIV has a worldwide distribution, and seropositive dairy and beef cattle have been recorded in many countries, including the United States (Cockerell et al., 1992), Canada (McNab et al., 1994), Iran (Nikbakht Brujeni et al., 2010; Tajbakhsh et al., 2010), Germany (Muluneh, 1994), France (Polack et al., 1996), Switzerland (Gene, 1994), Australia (Forman et al., 1992), the United Kingdom (Clayton, 1994), Japan (Hirai et al., 1996; Meas et al., 1998), Italy (Cavirani et al., 1998), Korea (Cho et al., 1999), Mexico (González‐Fernández et al., 2020), India (Patil et al., 2003) and Brazil (Meas et al., 2002; Rodrigues et al., 2019). However, very few publications can be found in the literature addressing the prevalence of the virus in buffaloes (Albernaz et al., 2015; Bhatia et al., 2006; Meas, Ohashi, et al., 2000; Meas, Seto, et al., 2000).

The domestic buffalo (*Bubalus bubalis*), also known as Asian or water buffalo, plays an important role in the economy of many countries (Minervino et al., 2020). Buffalo breeding has been developed in Iran since 2500 B.C. (Safari et al., 2018). They have been reared as a source of milk, meat and draft power. It is estimated that 480,000 heads of water buffalos live in Iran. Moreover, 16% of buffaloes in Iran are slaughtered, which yields a weight of approximately 12,960 tons of meat per year. It is assumed that the economic value of buffalo breeding in Iran is approximately equal to that of Holstein dairy cows. Nevertheless, to the authors’ best knowledge, no investigation has considered the circulating BIV strains in Iranian buffalo populations to date.

Two approaches based on serological methods and molecular techniques are often used to detect antibodies and the proviral genome of BIV, respectively. PCR (Polymerase Chain Reaction) as a molecular diagnostic technique is more sensitive than serological tests to detect and compare the DNA sequences to each other. However, direct amplification of BIV from blood samples is difficult and often leads to failure (Tajbakhsh et al., 2010). Hence, the most available results about BIV are obtained using serological methods.

The objectives of this study are as follows: First, to estimate the presence of BIV in buffalo populations in Iran for the first time. Second, to analyse the molecular and phylogenetic relationship of the positive isolates with BIV sequences recorded from other countries so far.

## 2 MATERIALS AND METHODS

### 2.1 Samples

A total of 120 blood samples were collected from water buffaloes (under 3 years of age) in the Khuzestan slaughterhouse located in southwest Iran. The samples were taken randomly from the animals transported to the slaughterhouse from traditional farms located in different regions of the southwest of the country. These samples were transported to the virology laboratory of the Veterinary faculty of Shiraz University in EDTA tubes and stored at −20°C until further processing.

### 2.2 DNA extraction

The buffy coat containing peripheral blood mononuclear cells was separated from red blood cells and plasma by centrifugation. The total genomic DNA of purified leukocytes was extracted using a Genomic DNA extraction kit (Genet Bio.) according to the manufacturer's instructions. Subsequently, the DNA concentration and purity were measured at the wavelength of 260 nm and 260/280 ratio, respectively, using a NanoDrop One^C^ Spectrophotometer (ThermoFisher Scientific).

### 2.3 Polymerase chain reaction and electrophoresis

To confirm the presence of BIV proviral DNA, all of the extracted DNA samples were searched for a 298‐bp fragment of *pol* gene sequence by nested PCR technique. Moreover, a recombinant plasmid containing the *pol* sequence (a kind gift from Prof. Gholamreza Nikbakht Brujeni) and a sample without the target sequence were considered as positive and negative controls, respectively. Two pairs of inner and outer primers suggested by Meas et al. (1998) were used for the polymerisation. The reaction was carried out in a 20 μl total reaction volume containing 10 μl of Taq DNA Polymerase 2x Master Mix (Amplicon), 1 μl of each 10 μM primer, 60 ng of DNA for the first reaction and 1 μl from the first round as template for the second round. The thermal cycling conditions for the first and second rounds of PCR were similar, consisting of initial denaturation at 94°C for 3 min, followed by 35 cycles of denaturation at 94°C for 30 s, annealing at 58°C for 45 s, extension at 72°C for 30 s and final extension at 72°C for 7 min. Finally, the amplified products were gel separated using 2% agarose. A plasmid containing the complete BIV pol coding region was used as a positive control (kindly gift from Prof. Gholamreza Nikbakht Brujeni).

### 2.4 Sequencing

The final 298‐bp fragments obtained from two samples resulting from the inner reaction were gel purified using an S‐1050‐1 DNA extraction kit (Dena zist) following the manufacturer's guidelines. Purified products of two PCR‐positive samples were sequenced in both directions using Sanger sequencing by Macrogen Company.

### 2.5 Bioinformatics analyses

The 298‐bp sequenced data were evaluated and edited using Geneious Prime (version 2019.1.3; http://www.geneious.com). The sequences that belong to the *pol* gene of BIV then were verified by the BLAST (Basic Local Alignment Search Tool) function of the National Center for Biotechnology Information database. Nucleotide sequences were deposited in the GenBank database under the accession numbers MZ574134 and MZ574135.

To further investigate the evolutionary relationship among available BIVs, a phylogenetic tree was generated based on the 298‐bp nucleotide conserved fragment of the *pol* gene. The MEGA 7 (version 7.0.26) program (Kumar et al., 2016), set at Maximum Likelihood, T92+I model (based on the best fit model suggested by MEGA7 algorithm), and 1000 bootstrapping was used to construct the phylogenetic tree. The studied sequences were compared with the related sequences for four other known BIV strains available in GenBank. Three HIVs with large distances were used as outgroups. Indeed, another phylogenetic tree was constructed based on a shorter sequence of the *pol* gene (163 bp) reported in other relevant studies (Supplementary Information 1).

Subsequently, all the available BIV strains were subjected to homology and sequence composition analyses in Geneious Prime (version 2019.1.3).

## 3 RESULTS

Among 120 independent peripheral blood samples tested for the presence of BIV, 11 (9.1%) specimens were positive by nested PCR. As shown in Table 1, the studied isolates shared 100% identity with each other. Indeed, they exhibited high nucleotide homology of more than 98%–99% to other BIV sequences deposited in GenBank to date (Table 1). The purine transitions were identified among isolates described in Table 2. A BIV R29 strain (L04974) with two amino acid substitutions had the maximum distance to the studied sequences (Table 2).

**TABLE 1:** Nucleotide (upper half) and amino acid (lower half) sequence identity matrix for the studied isolates and other bovine immunodeficiency viruses (BIVs) based on pol

	**MZ574134**	**MZ574135**	**NC_0014133**	**L04972**	**M32690**	**L04974**
MZ574134^a^		100	99.33	98.66	99.33	99.67
MZ574135^a^	100		99.33	98.66	99.33	99.67
NC_001413	98.99	98.99		99.33	100	99.00
L04972	97.98	97.98	98.99		99.33	98.33
M32690	98.99	98.99	100	98.99		99.00
L04974	98.99	98.99	97.98	96.97	97.98	

^a^ The studied Iranian isolates.

**TABLE 2:** Comparison of nucleotide and amino acid sequences of the *pol* gene of the studied strains with other BIVs

	**Nucleotide position**			**Amino acid position**		
**Strain**	**2245**	**2248**	**2272**	**225**	**226**	**234**
MZ574134^a^	G	G	A	G	R	K
MZ574135^a^	G	G	A	G	R	K
L04972	G	A	G	G	K	R
L04974	A	G	A	E	R	K
NC_001413	G	G	G	G	R	R
M32690	G	G	G	G	R	R

^a^ The studied Iranian strains.

**FIGURE 1:**
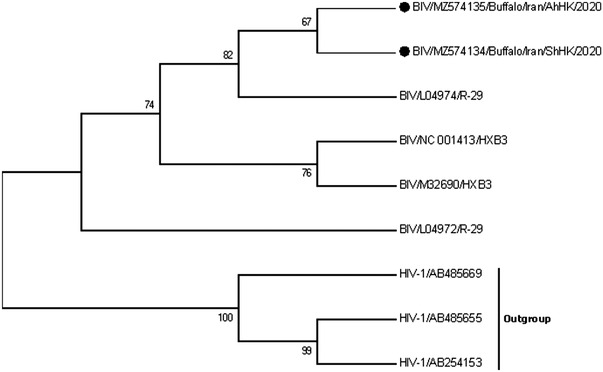
Phylogenetic tree of the nucleotide sequences of the 298‐bp coding region of the *pol* gene of the studied isolates and other related BIV sequences. The maximum likelihood method and T92+I model with 1000 bootstrap replicates were used. The isolated subject of the current study is marked by a circle. BIV, bovine immunodeficiency virus; HIV‐1, human immunodeficiency virus type I

The phylogenetic tree placed the BIVs in a unique cluster closely related to each other (Figure 1).

## 4 DISCUSSION

The main concern of the paper is to draw attention to BIV's importance in buffalo populations. To our knowledge, this is the first study to investigate the prevalence of BIV in buffaloes in Iran. In this regard, the authors focused not only on the occurrence of the infection in buffalo populations in southwestern Iran but also on the phylogenetic relationship of the studied isolates that were compared to other sequences previously reported.

The prevalence of 9.1% is more similar to the reports from buffaloes in Pakistan (10.3%; Meas, Seto, et al., 2000). However, two other published studies from Brazil and Cambodia suggested lower (4.4%) and higher (16.7%) incidences of BIV in buffaloes, respectively (Albernaz et al., 2015; Meas, Ohashi, et al., 2000). While it seems that buffaloes are more resistant to infections, they can transmit BIV to more susceptible cattle. Moreover, detection of the virus in animals even without obvious clinical manifestations has negative impacts on productivity and predisposes them to secondary infections (Mahzounieh et al., 2013; Nikbakht Brujeni et al., 2010).

The occurrence of BIV in cattle follows a non‐uniform distribution in Iran: 20.3% in Tehran (Nikbakht Brujeni et al., 2010), 60% (Tajbakhsh et al., 2010) and 5.7% (Meas et al., 2003) in Chaharmahale Bakhtiary (southwestern part of the country) and 1.12% in Isfahan, central Iran (Meas et al., 2003). Furthermore, the seroprevalence of the infection in Asian countries has been studied. Two studies conducted in Japan revealed collective 7.5% (Hirai et al., 1996) and 11.7% (Meas et al., 1998) seropositivities in different cattle herds. In Korea, 35% and 33% of dairy and beef cattle, respectively, were positive for anti‐BIV antibodies (Cho et al., 1999). Meas et al. detected the BIV‐provirus DNA in all (12.3%) seropositive samples obtained from four cattle herds in Turkey (Horzinek et al., 1991). Patil et al. (2003) proposed that the proviral genomic sequence of BIV was detected in 8.8% of Indian cattle. More seropositive cases have also been reported from the United Kingdom (Clayton, 1994), Canada (McNab et al., 1994), Germany (Muluneh, 1994), France (Polack et al., 1996), Australia (Forman et al., 1992), the Netherlands (Meas et al., 2004), the United States (Cockerell et al., 1992), Zambia (Jacobs et al., 1998), Mexico (Meas et al., 1998) and Italy (Cavirani et al., 1998). The incidence rate of infection in European countries was lower than that in Asian regions.

Although several studies have used serological tests for the detection of BIV‐specific antibodies so far, PCR as a direct method provides a more precise diagnosis. Also, the subsequent data obtained from the sequencing helped us to discover the evolutionary relationship among isolates (Gonzalez et al., 2001). Nested PCR can detect low copy numbers of the target sequences, decreasing the false‐negative rates (Meas et al., 2001). In this regard, *pol*, as a conserved gene, was used for molecular and phylogenetic analyses. Studies on *pol* are still lacking, and already available data from Iran were performed based on the *gag* gene in cattle. However, we compared our sequences with four available isolates from other countries deposited in GenBank. The studied isolates had more than 98%–99% nucleotide identities with R‐29 references and HXB3 sequences (Table 1). The results thus obtained are compatible with the findings of some other studies (Albernaz et al., 2015; Jacobs et al., 1998; Meas et al., 2002, 2003; Rodrigues et al., 2019).

Genomic variability is a significant feature of RNA viruses, including BIV. Even within a single virus isolate, BIV exhibits considerable genomic variation (Esmailnejad et al., 2020; Garvey et al., 1990). However, pol is a conserved sequence, and as an intracellular protein, it endures less immune selective pressure. In this regard, our sequence comparisons did not show extensive variation among the different strains shown in Table 2. Three nucleotide changes were observed among BIV isolates (G→A or A→G), all of which caused amino acid substitutions at positions 225, 226 and 234 (Table 2). Nevertheless, the studied isolates were completely identical to each other.

BIV has several accessory genes, including viral infectivity factor, trans‐activator factor of transcription, regulator of virus expression, *vpw*, *vpy* and *tmx*, which are responsible for the regulation of gene expression. However, DNA sequence variation has mainly been detected in the three major ORFs (Open Reading Frames) of BIV encoding the *gag*, *env* and *pol* genes. The *env* gene in retroviruses undergoes frequent selective pressure so that it is implemented for molecular epidemiology studies (González‐Fernández et al., 2020). This genomic variability is in part due to the infidelity of the reverse transcriptase of the virus during replication, which is supposed to form quasi‐species populations of the virus (Garvey et al., 1990).

Despite the genetic diversity among different retroviruses, the amino‐terminal end of pol is known as a conserved domain that can be used for generating phylogenetic trees and comparing the evolutionary relationship of isolates. Figure 1 shows different BIVs in a unique main cluster. Variants diverged from an unknown common strain and then separated from each other over time. Generally, the number of available *pol* sequences in nucleic acid databases is limited, and these findings do not enable us to determine a conclusive remark. Moreover, the limited number of the studied samples as well as the small size of the amplicons can be the cause of the little variation observed among sequences. However, the findings advance our understanding of new sequences and could be useful for further research.

The rate of BLV infection in the studied buffalo population was checked in our previous experiment (González et al., 2008). Despite the study conducted by Nikbakht Brujeni et al. (2010) conducted in Holstein cattle, the total numbers of BLV‐positive samples (52%) were greater than the BIV cases (9.1%) in our experiments. However, based on some other studies (Cho et al., 1999; Clayton, 1994; Horzinek et al., 1991; McNab et al., 1994; Meas, Ohashi, et al., 2000; Nikbakht Brujeni et al., 2010), incidental mixed infection of dams with both BIV and BLV may relate to the similar modes of transmission of the infections through blood transfusion, insemination, and contaminated instruments. However, González et al. (2008) and Meas, Seto, et al. (2000) found some evidence confirming the positive correlation of dual infection of cattle with BIV and BLV.

The relation between BIV and other secondary infections such as BLV, the Jembrana disease, and the bovine syncytial virus is a question still unanswered and can be considered in future work. Genetic and environmental factors are critical criteria affecting the prevalence and severity of the disease. However, more research is still necessary before obtaining a definitive answer to explain the cause of the wide range of BIV incidence (1%–60%) recorded in Iran. Indeed, further research on the effects of nucleotide changes on protein properties and the routes of transmission of the infection is desirable to extend our knowledge of disease management.

## 5 CONCLUSION

In conclusion, the present study clearly shows that the buffalo populations in southwestern Iran are infected with BIV. The incidence rate of the infection obtained from this study (9.1%) is inconsistent with previous records from cattle herds in other parts of Iran. However, our isolates were completely identical to each other and had high nucleotide homology of more than 98%–99% to sequences previously reported from other countries. Since not many molecular studies have been performed on BIV sequences in the world, we could not have a comprehensive interpretation of the origin and evolutionary process of the virus. However, this study, as the first investigation of BIV in the buffalo population in Iran, can be used for performing further research to prevent and control the disease.

## CONFLICT OF INTEREST

The authors declare no conflict of interest.

## AUTHOR CONTRIBUTIONS

*Conceptualisation, validation, resources, data curation, review and editing*: Ali Mohammadi. *Validation, formal analysis, investigation*: Haniyeh Keshavarz. *Writing original draft, review and editing*: Solmaz Morovati. All authors have read and agreed to the published version of the manuscript.

## ETHICS STATEMENT

This research was approved by the Iranian Society for the Prevention of Cruelty to Animals and Shiraz University Research Council (IACUC no: 4687/63). Also, the recommendations of the European Council Directive (2010/63/EU) of September 22, 2010, regarding the standards in the protection of animals used for experimental purposes were also followed.

### PEER REVIEW

The peer review history for this article is available at https://publons.com/publon/10.1002/vms3.872.

## Supporting information

Supplementary Information 1. Phylogenetic tree of the nucleotide sequences of the 163‐bp coding region of the *pol* gene of the studied isolates and other related BIV sequences. The maximum likelihood method and T92+I model with 1000 bootstrap replicates were used. The isolated subject of the current study is marked by a circle.

## Data Availability

All data used and analysed during the current study are available from the corresponding author upon reasonable request.

## References

[vms3872-bib-0001] Albernaz, T. T. , Leite, R. C. , Reis, J. K. P. , De Sousa Rodrigues, A. P. , Da Cunha Kassar, T. , Resende, C. F. , De Oliveira, C. H. S. , Silva, R. D. M. , Salvarani, F. M. , & Barbosa, J. D. (2015). Molecular detection of bovine immunodeficiency virus in water buffaloes (*Bubalus bubalis)* from the Amazon region, Brazil. Tropical Animal Health and Production, 47(8), 1625–1628. 26174574 10.1007/s11250-015-0884-6

[vms3872-bib-0002] Bhatia, S. , Bhatia, A. , Sood, R. , Pattnaik, B. , & Pradhan, H. (2006). Serological evidence of bovine immunodeficiency virus infection in cattle and buffalo through use of recombinant capsid (P26) protein based immunoassay. Journal of Immunology and Immunopathology, 8(2), 128–129.

[vms3872-bib-0003] Cavirani, S. , Donofrio, G. , Chiocco, D. , Foni, E. , Martelli, P. , Allegri, G. , Cabassi, C. S , De Iaco, B. , & Flammini, C. F. (1998). Seroprevalence to bovine immunodeficiency virus and lack of association with leukocyte counts in Italian dairy cattle. Preventive Veterinary Medicine, 37(1‐4), 147–157. 9879588 10.1016/s0167-5877(98)00099-3

[vms3872-bib-0004] Cho, K. ‐O. , Meas, S. , Park, N. ‐Y. , Kim, Y. ‐H. , Lim, Y. ‐K. , Endoh, D. , Lee, S. ‐I. , Pjasjo, K. , Sugimoto, C. , & Onuma, M. (1999). Seroprevalence of bovine immunodeficiency virus in dairy and beef cattle herds in Korea. Journal of Veterinary Medical Science, 61(5), 549–551. 10379949 10.1292/jvms.61.549

[vms3872-bib-0005] Clayton, J. (1994). Spectre of AIDS haunts reports of sick cows. Nature, 367(6464), 585. 10.1038/367585a0 8107834

[vms3872-bib-0006] Cockerell, G. L. , Jensen, W. A. , Rovnak, J. , Ennis, W. H. , & Gonda, M. A. (1992). Seroprevalence of bovine immunodeficiency‐like virus and bovine leukemia virus in a dairy cattle herd. Veterinary Microbiology, 31(2‐3), 109–116. 1320785 10.1016/0378-1135(92)90069-6

[vms3872-bib-0007] Esmailnejad, A. , Najafi, H. , & Torfi, Y. (2020). Molecular and serological evaluation of bovine leukemia virus in water buffaloes of southern Iran. Iranian Journal of Veterinary Medicine, 14(1), 37–44.

[vms3872-bib-0008] Forman, A. , Gibson, C. , & Rodwell, B. (1992). Serological evidence for the presence of bovine lentivirus infection in cattle in Australia. Australian Veterinary Journal, 69(12), 337–337. 10.1111/j.1751-0813.1992.tb09919.x 1337246

[vms3872-bib-0009] Garvey, K. J. , Oberste, M. S , Elser, J. E. , Braun, M. J. , & Gonda, M. A. (1990). Nucleotide sequence and genome organization of biologically active proviruses of the bovine immunodeficiency‐like virus. Virology, 175(2), 391–409. 2183467 10.1016/0042-6822(90)90424-p

[vms3872-bib-0010] Gene, L. D. (1994). Bovine immunodeficiency virus: molecular biology and virus‐host interactions. Virus Research, 32(2), 155–181. 8067052 10.1016/0168-1702(94)90040-x

[vms3872-bib-0011] Gonda, M. A. , Braun, M. J. , Carter, S. G. , Kost, T. A. , Bess, J. W. , Arthur, L. O. , & Maaten, M. J. V. D. (1987). Characterization and molecular cloning of a bovine lentivirus related to human immunodeficiency virus. Nature, 330(6146), 388–391. 3683555 10.1038/330388a0

[vms3872-bib-0012] González, E. T. , Licursi, M. , Vila Roza, V. , Bonzo, E. , Mortola, E. , Frossard, J. P. , & Venables, C. (2008). Evidence of bovine immunodeficiency virus (BIV) infection: Serological survey in Argentina. Research in Veterinary Science, 85(2), 353–358. 18037459 10.1016/j.rvsc.2007.10.008

[vms3872-bib-0013] Gonzalez, G. , Johnston, J. , Nickel, D. , Jacobs, R. , Olson, M. , & Power, C. (2001). Very low prevalence of bovine immunodeficiency virus infection in western Canadian cattle. Canadian Journal of Veterinary Research, 65(1), 73. 11227201 PMC1189647

[vms3872-bib-0014] González‐Fernández, V. D. , Tortora Perez, J. L. , Garcia Flores, M. M. , Aguilar Setien, J. A. , & Ramirez Alvarez, H. (2020). First evidence of bovine immunodeficiency virus infection in Mexican cattle. Transboundary and Emerging Diseases, 67(5), 1768–1775. 10.1111/tbed.13530 32129921

[vms3872-bib-0015] Hirai, N. , Kabeya, H. , Ohashi, K. , Sugimoto, C. , & Onuma, M. (1996). Detection of antibodies against bovine immunodeficiency‐like virus in daily cattle in Hokkaido. Journal of Veterinary Medical Science, 58(5), 455–457. 8741608 10.1292/jvms.58.455

[vms3872-bib-0016] Horzinek, M. , Keldermans, L. , Stuurman, T. , Black, J. , Herrewegh, A. , Sillekens, P. , & Koolen, M. (1991). Bovine immunodeficiency virus: Immunochemical characterization and serological survey. Journal of General Virology, 72(12), 2923–2928. 1722502 10.1099/0022-1317-72-12-2923

[vms3872-bib-0017] Jacobs, R. M. , Jefferson, B. J. , & Suarez, D. L. (1998). Prevalence of bovine immunodeficiency‐like virus in bulls as determined by serology and proviral detection. Canadian Journal of Veterinary Research, 62(3), 231. 9684054 PMC1189481

[vms3872-bib-0018] Kumar, S. , Stecher, G. , & Tamura, K. (2016). MEGA7: molecular evolutionary genetics analysis version 7.0 for bigger datasets. Molecular Biology and Evolution, 33(7), 1870–1874. 27004904 10.1093/molbev/msw054 PMC8210823

[vms3872-bib-0019] Mahzounieh, M. , Mokhtari, A. , & Pierre, F. J. (2013). Serologic and molecular studies on prevalence of bovine immunodeficiency virus (BIV) infection in cattle in Chahrmahal va Bakhtiari and Isfahan provinces. Journal of Veterinary Microbiology, 8(2), 91–100.

[vms3872-bib-0020] McNab, W. B. , Jacobs, R. M. , & Smith, H. E. (1994). A serological survey for bovine immunodeficiency‐like virus in Ontario dairy cattle and associations between test results, production records and management practices. Canadian Journal of Veterinary Research, 58(1), 36. 8143251 PMC1263657

[vms3872-bib-0021] Meas, S. , Kabeya, H. , Yoshihara, S. , Ohashi, K. , Matsuki, S. , Mikami, Y. , Sugimoto, C. , & Onuma, M. (1998). Seroprevalence and field isolation of bovine immunodeficiency virus. Journal of Veterinary Medical Science, 60(11), 1195–1202. 9853299 10.1292/jvms.60.1195

[vms3872-bib-0022] Meas, S. , Nakayama, M. , Usui, T. , Nakazato, Y. , Yasuda, J. , Ohashi, K. , & Onuma, M. (2004). Evidence for bovine immunodeficiency virus infection in cattle in Zambia. Japanese Journal of Veterinary Research, 52(1), 3–8. 15253302

[vms3872-bib-0023] Meas, S. , Ohashi, K. , Sugimoto, C. , & Onuma, M. (2001). Phylogenetic relationships of bovine immunodeficiency virus in cattle and buffaloes based on surface envelope gene sequences. Archives of Virology, 146(5), 1037–1045. 11448023 10.1007/s007050170134

[vms3872-bib-0024] Meas, S. , Ohashi, K. , Tum, S. , Chhin, M. , Te, K. , Miura, K. , Sugimoto, C. , & Onuma, M. (2000). Seroprevalence of bovine immunodeficiency virus and bovine leukemia virus in draught animals in Cambodia. Journal of Veterinary Medical Science, 62(7), 779–781. 10945301 10.1292/jvms.62.779

[vms3872-bib-0025] Meas, S. , Ruas, F. J. , Farias, N. A. , Usui, T. , Teraoka, Y. , Mulenga, A. , Chang, K. ‐S. , Masuda, A. , Madruga, C. R. , Ohashi, K. , & Onuma, M. (2002). Seroprevalence and molecular evidence for the presence of bovine immunodeficiency virus in Brazilian cattle. Japanese Journal of Veterinary Research, 50(1), 9–16. 12201018

[vms3872-bib-0026] Meas, S. , Seto, J. , Sugimoto, C. , Bakhsh, M. , Riaz, M. , Sato, T. , Naeem, K. , Ohashi, K. , & Onuma, M. (2000). Infection of bovine immunodeficiency virus and bovine leukemia virus in water buffalo and cattle populations in Pakistan. Journal of Veterinary Medical Science, 62(3), 329–331. 10770609 10.1292/jvms.62.329

[vms3872-bib-0027] Meas, S. , Yilmaz, Z. , Usui, T. , Torun, S. , Yesilbag, K. , Ohashi, K. , & Onuma, M. (2003). Evidence of bovine immunodeficiency virus in cattle in Turkey. Japanese Journal of Veterinary Research, 51(1), 3–8. 12921344

[vms3872-bib-0028] Minervino, A. H. H. , Zava, M. , Vecchio, D. , & Borghese, A. (2020). *Bubalus bubalis*: A short story. Frontiers in Veterinary Science, 7, 971. 10.3389/fvets.2020.570413 PMC7736047 33335917

[vms3872-bib-0029] Muluneh, A. (1994). Seroprevalence of bovine immunodeficiency‐virus (BIV) antibodies in the cattle population in Germany. Journal of Veterinary Medicine, Series B, 41(1‐10), 679–684. 7597862 10.1111/j.1439-0450.1994.tb00280.x

[vms3872-bib-0030] Nikbakht Brujeni, G. , Taghi Poorbazargani, T. , Nadin‐Davis, S. , Tolooie, M. , & Barjesteh, N. (2010). Bovine immunodeficiency virus and bovine leukemia virus and their mixed infection in Iranian Holstein cattle. Journal of Infection in Developing Countries, 4(09), 576–579. 21045371 10.3855/jidc.711

[vms3872-bib-0031] Patil, S. , Pattnaik, B. , Mishra, N. , Banumathi, N. , Dubey, R. , & Pradhan, H. (2003). Detection of proviral genomic sequence of bovine immunodeficiency virus in Indian cattle. Current Science, *84*(4), 563–566.

[vms3872-bib-0032] Polack, B. , Schwartz, I. , Berthelemy, M. , Belloc, C. , Manet, G. , Vuillaume, A. , Baron, T. , Gonda, M. A. , & Lévy, D. (1996). Serologic evidence for bovine immunodeficiency virus infection in France. Veterinary Microbiology, 48(1‐2), 165–173. 8701572 10.1016/0378-1135(95)00138-7

[vms3872-bib-0033] Rodrigues, A. P. S. , Fonseca Júnior, A. A. , Lima, G. K. , Bicalho, J. M. , Leite, R. C. , & Reis, J. K. P. (2019). Molecular detection of bovine immunodeficiency virus (BIV) in bovines from the state of Minas Gerais, Brazil. Arquivo Brasileiro de Medicina Veterinária e Zootecnia, 71(2), 711–714.

[vms3872-bib-0034] Safari, A. , Ghavi Hossein‐Zadeh, N. , Shadparvar, A. A. , & Abdollahi Arpanahi, R. (2018). A review on breeding and genetic strategies in Iranian buffaloes (*Bubalus bubalis*). Tropical Animal Health and Production, 50(4), 707–714. 29524107 10.1007/s11250-018-1563-1

[vms3872-bib-0035] Tajbakhsh, E. , Borujeni, G. N. , Momtazan, H. , & Amirmozafari, N. (2010). Molecular prevalence for bovine immunodeficiency virus infection in Iranian cattle population. African Journal of Microbiology Research, 4(12), 1199–202.

